# Supplementary material for: The effects of familiarity on escape responses in the Trinidadian guppy (Poecilia reticulata)
Source: PeerJ. 2017 Oct 11;5:e3899. doi: 10.7717/peerj.3899 (PMC5640977; doi:10.7717/peerj.3899)
Supplement: Data S1 [file peerj-05-3899-s004.docx]

The raw data has been supplied as supplementary File.

http://dx.doi.org/10.17630/92831d81-38f0-4573-b2e5-e1d11adf9322
